# Selective Appendectomy in Patients Undergoing Minimally Invasive Surgery for Endometriosis: A Retrospective Cohort Study

**DOI:** 10.3390/jcm14207277

**Published:** 2025-10-15

**Authors:** Camran Nezhat, Zahra Najmi, Maryam Mirzaie, Quincy Harding, Zoë Pennington, Nikki Amirlatifi, Rana Khaloghli, Dahnia Zarroug, Eric R. Sokol

**Affiliations:** 1 Camran Nezhat Institute Center for Minimally Invasive and Robotic Surgery, Woodside, CA 94061, USA; 2 Stanford University Medical Center, Palo Alto, CA 94305, USA; 3 University of California, San Francisco, San Francisco, CA 94143, USA; 4 California State Polytechnic University, Pomona, CA 91768, USA; 5 Stanford University, Palo Alto, CA 94305, USA; 6 Urogynecology and Reconstructive Pelvic Surgery, School of Medicine, Stanford University, Stanford, CA 94305, USA; esokol@stanford.edu

**Keywords:** endometriosis, appendiceal pathology, appendiceal endometriosis, laparoscopic surgery, minimally invasive surgery, selective appendectomy, chronic pelvic pain, neuroendocrine tumor, appendix obliteration, adhesion, inflammation, histopathology, robotic assisted surgery

## Abstract

**Background/Objectives:** Endometriosis is a chronic inflammatory systemic disease that commonly affects bowel structures, including the appendix, where it may mimic or coexist with chronic appendicitis. Visual inspection alone often fails to detect appendiceal involvement, leading to underdiagnosis and suboptimal management. This study investigates the prevalence and histopathologic spectrum of appendiceal abnormalities in patients undergoing minimally invasive laparoscopic surgery for endometriosis and evaluates the safety and postoperative outcomes of selective appendectomy. **Methods:** We conducted a retrospective cohort study of 236 patients who underwent a selective appendectomy concurrent with laparoscopic surgery for endometriosis with and without robotic assistance from January 2024 to April 2025. Preoperative evaluation included clinical assessment, imaging, and risk stratification using the Nezhat Endometriosis Risk Advisor tool, with some patients referred after positive ReceptivaDx testing. Intraoperatively, the appendix was examined for endometriosis, adhesions, or obliteration, and abnormal findings warranted removal using a vascular stapler. Postoperative outcomes and histopathologic results were assessed over six months, with appendiceal involvement analyzed in relation to endometriosis stage. **Results:** Of 236 patients who underwent selective laparoscopic appendectomy during surgical treatment for endometriosis, abnormal appendiceal pathology was identified in 216 (91.53%) patients. Histopathology revealed appendiceal endometriosis in 34 patients (14.41%), adhesions in 140 (59.32%), fibrous obliteration in 82 (34.75%), inflammation in 20 (8.47%), and neuroendocrine tumors in 3 (1.27%), one of which was malignant. Endometriotic lesions of the appendix showed a significant association with advanced-stage (III–IV) disease (*p* = 0.05), while other pathologies were not stage-dependent. No intraoperative complications occurred, and postoperative outcomes were favorable, with only one readmission unrelated to the appendectomy. **Conclusions:** Selective appendectomy during laparoscopic surgery for endometriosis revealed a high prevalence (91.5%) of appendiceal pathology. Even without visible implants, the appendix may contribute to symptoms, underscoring the importance of thorough intraoperative evaluation. Selective appendectomy based on surgical findings may aid symptom relief, prevent missed diagnoses, and enhance comprehensive management of endometriosis, but these potential benefits must be weighed against the small risks of concurrent appendectomy.

## 1. Introduction

Endometriosis is a chronic systemic inflammatory disease characterized by endometrial-like tissue present ectopically, particularly in the pelvic area [1,2]. The pathophysiology of the disease involves elevated estrogen levels, impaired progesterone signaling, inflammation in the eutopic endometrium, and ectopic fibrosis development. Patients with endometriosis experience various symptoms including secondary dysmenorrhea, pelvic pain, pain at ovulation (mid-cycle), and/or dyspareunia [3]. Endometriosis is the most common cause of unexplained infertility [4]. Failure to treat before conceiving could result in higher rates of miscarriage, ectopic pregnancy, preterm birth, bleeding, and placental abnormalities [5]. Endometriosis lesions can spread to neighboring organs and consequently lead to inflammation, pain, fibrosis, and in severe cases, organ malfunction including silent kidney loss and bowel obstruction [3,4,5,6,7].

Bowel involvement is considered one of the most significant and challenging manifestations of endometriosis. The prevalence of intestinal endometriosis, including the appendix, is reported between 3.8% and 37%, with this variation attributed to differences in classification and potential instances of underdiagnosis. Bowel endometriosis warrants careful consideration in differential diagnoses due to its prevalence and the potential consequences of misdiagnosis. Delays in accurate identification can prolong the time to effective management, adversely affecting patients’ quality of life [7].

The prevalence of appendiceal involvement in endometriosis is reported to range between 2.6% and 13.2%, depending on the study design and whether the appendix is systematically evaluated during surgery. In a comprehensive review and case series by Gustofson et al., appendiceal endometriosis was identified in 13.2% of women undergoing laparoscopic surgery for endometriosis, and notably, many of these cases had a grossly normal-appearing appendix, highlighting the limitations of relying on visual inspection alone [8]. Similarly, a retrospective study by Centini et al. reported appendiceal endometriosis in 2.8% out of 460 patients affected by endometriosis, suggesting that the true prevalence may be underestimated when the appendix is not routinely examined or excised [9]. These findings underscore the importance of careful intraoperative evaluation of the appendix, particularly in patients with chronic pelvic pain or deep infiltrating endometriosis.

Chronic appendicitis is a rare and often debated clinical entity characterized by prolonged or recurrent inflammation of the appendix, typically presenting with vague, intermittent right lower quadrant abdominal pain and sometimes accompanied by mild gastrointestinal symptoms such as nausea, bloating, or changes in bowel habits. Unlike acute appendicitis, it lacks the classic signs of acute infection and often presents with normal or minimally elevated inflammatory markers, making diagnosis challenging. Imaging may reveal subtle findings such as wall thickening or appendiceal distension without signs of rupture, while histopathologic evaluation after appendectomy may demonstrate chronic inflammatory infiltrates, fibrosis, or lymphoid hyperplasia [10]. While chronic appendicitis can be a source of chronic pelvic pain, it can clinically be confused with endometriosis due to overlapping features such as nonspecific gastrointestinal symptoms, inconclusive imaging, and similar patterns of pelvic pain, especially in reproductive-aged women. Endometriosis involving the appendix or adjacent pelvic structures can mimic chronic appendicitis by causing localized inflammation, adhesions, or fibrosis. Conversely, chronic appendicitis may resemble the cyclic or chronic pelvic pain of endometriosis. Intraoperatively, both conditions may present with a thickened or inflamed appendix, necessitating histopathologic examination for a definitive diagnosis. Importantly, these conditions can coexist, further complicating clinical interpretation and emphasizing the need for careful intraoperative evaluation [8,10].

Performing an appendectomy selectively during endometriosis surgery has the benefits of removing possible appendiceal pathology, and eliminating the need for a possible future surgery [11]. Selective surgery allows for a single recovery period rather than two separate ones, while also reducing overall healthcare costs [12]. Removal of the appendix has an added benefit of reducing diagnostic ambiguity in future instances of acute abdominal pain [12].

In this study, we aim to investigate the prevalence of appendiceal abnormalities in patients undergoing laparoscopic surgery for endometriosis. We also assess the safety and postoperative outcomes of performing appendectomy selectively with endometriosis surgery. Additionally, we analyze the histopathologic findings of the removed appendices to better understand the spectrum of appendiceal pathologies that may be present in this patient population. Through this comprehensive approach, we aim to provide evidence to support informed surgical decision-making regarding the evaluation and management of the appendix during endometriosis procedures.

## 2. Materials and Methods

This retrospective cohort study included 236 patients who underwent laparoscopic appendectomy selectively with laparoscopic surgery, with or without robotic assistance, for the diagnosis and treatment of endometriosis between January 2024 and April 2025. This study was conducted in accordance with the West Coast Institutional Review Board (IRB protocol number: 20230726). All procedures were performed by the senior lead surgeon Dr. Camran Nezhat and his team of clinical fellows. The inclusion criterion was the performance of an appendectomy during laparoscopic endometriosis surgery. No patients were excluded once this criterion was met. The primary surgical indications were endometriosis-related symptoms, primarily chronic pelvic pain and/or infertility.

Preoperative diagnosis of endometriosis was based on a combination of clinical evaluation and imaging studies. All patients were initially screened using the Nezhat Endometriosis Risk Advisor application, a validated digital tool reported to have over 90% accuracy in identifying suspected endometriosis based on patient history and symptom profile [13]. Additional diagnostic workup included transvaginal ultrasound, magnetic resonance imaging (MRI), and pelvic examination. A subset of patients was referred following a positive ReceptivaDx test, indicating possible progesterone resistance or BCL6 overexpression, commonly associated with endometriosis-related infertility [14].

**Figure 1:**
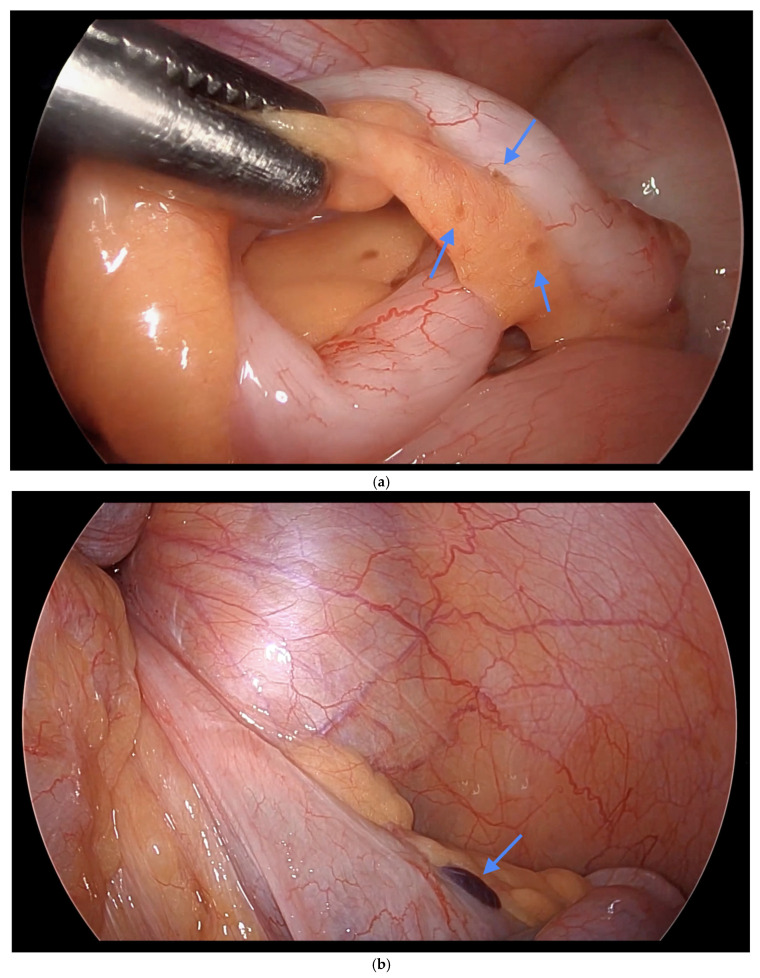
(**a**) Endometriosis on the appendix and mesoappendix. (**b**) Endometriosis nodule at the base of the appendix. Blue arrows indicate (**a**) endometriosis involving the appendix and mesoappendix, and (**b**) an endometriosis nodule at the base of the appendix.

**Figure 2:**
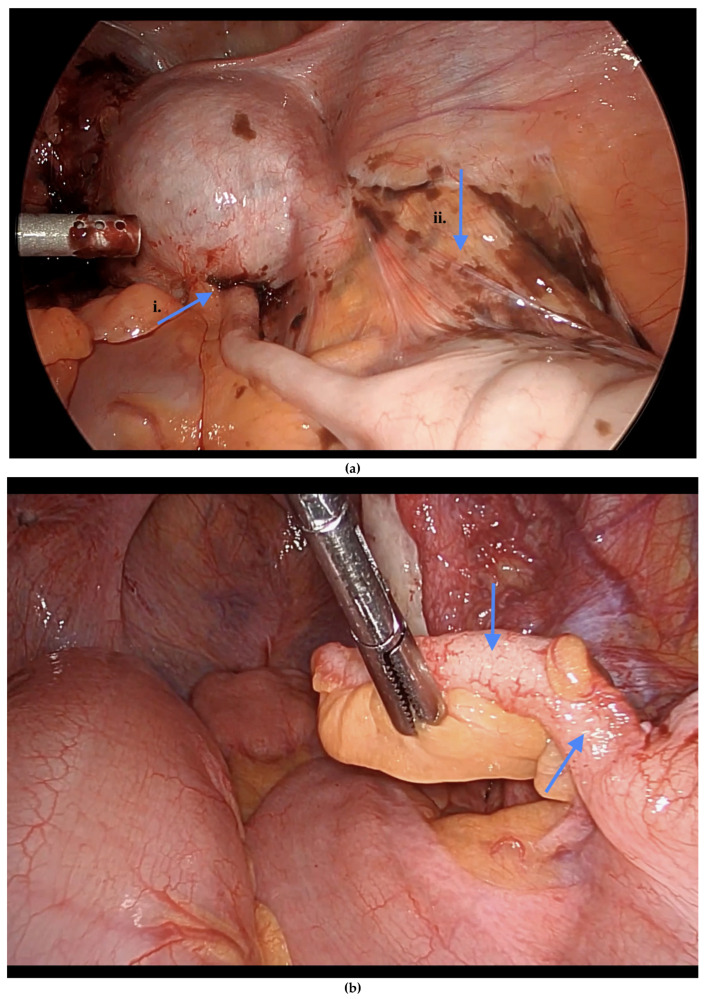
(**a**) i. Dense adhesions of the appendix to the uterus. ii. Endometriosis surrounding the peri appendix. (**b**) Vesicular lesions on the appendix. Blue arrows indicate (**a**) dense adhesions of the appendix to the uterus with endometriosis surrounding the peri-appendix, and (**b**) vesicular lesions on the appendix.

**Figure 3:**
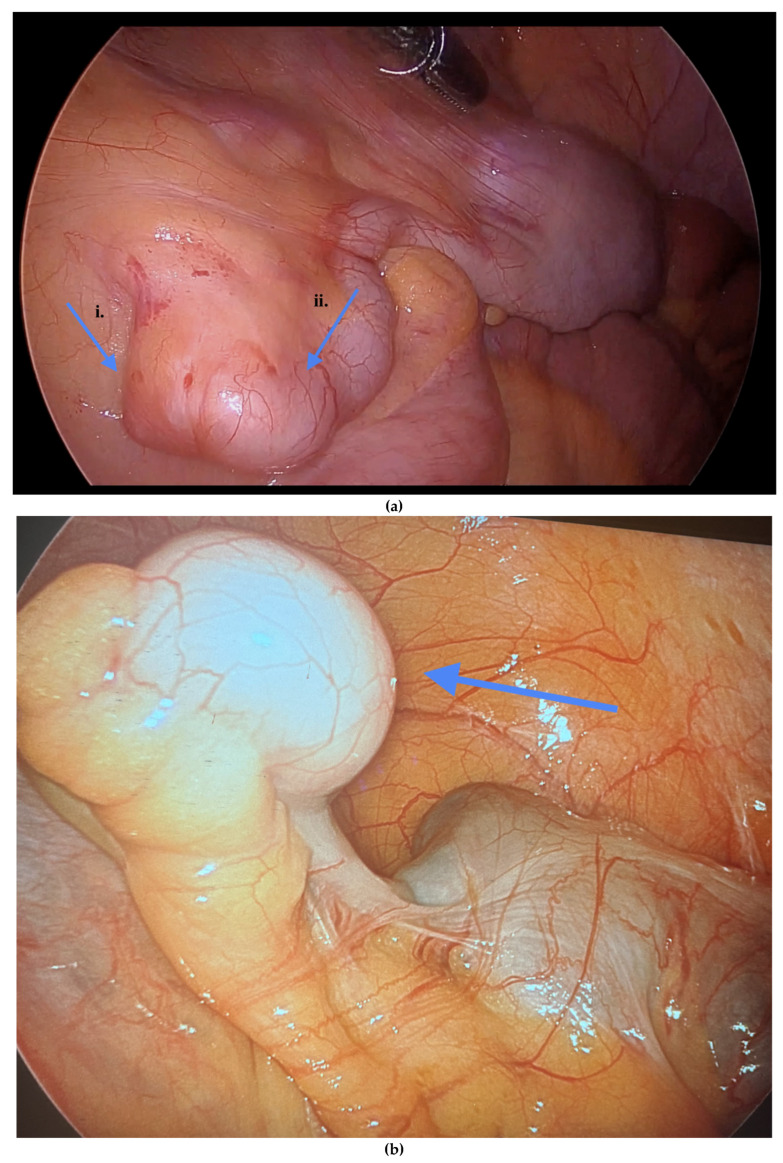
(**a**) i. Fibrous obliteration, and endometriosis at the tip. ii. Inflammation of the appendix indicating possible acute appendicitis. (**b**) Endometriosis on the tip of the appendix. Blue arrows indicate (**a**) (i) fibrous obliteration and endometriosis at the tip of the appendix, and (ii) inflammation suggesting possible acute appendicitis; and (**b**) endometriosis at the tip of the appendix.

Informed consent was obtained preoperatively, which included a discussion of the possibility of intraoperative appendectomy if appendiceal abnormalities were identified. During surgery, after optimal excision and ablation of endometriotic lesions, the appendix was carefully evaluated for evidence of endometriotic implants, serosal abnormalities, adhesions, fibrous obliteration, or features suggestive of acute or chronic appendicitis (Figure 1, Figure 2 and Figure 3). If abnormal findings were present, intravenous metronidazole was administered for infection prophylaxis, and a laparoscopic appendectomy was performed using the existing port sites [15]. The surgical technique included division of the mesoappendix, followed by transection and closure of the appendiceal base using a vascular stapler. The specimen was placed into a laparoscopic retrieval pouch and sent for histopathologic analysis [15,16].

Postoperative outcomes were evaluated over a six-month follow-up period through a combination of routine clinical assessments and patient self-reports. The primary outcome measures included the occurrence of intraoperative and postoperative complications: surgical site infection, postoperative bleeding, chronic pelvic pain, hospital readmission, or the need for reoperation. All excised appendiceal specimens were submitted for histopathological analysis. The study assessed the prevalence of abnormal histopathologic findings and further classified the types of appendiceal pathologies identified. Additionally, the distribution of these histopathologic abnormalities was analyzed in relation to the different stages of endometriosis, to determine any correlation between disease severity and appendiceal involvement.

The classification of endometriosis severity in this study was based on a recently updated staging system. This revised method categorizes endometriosis into genital and extragenital stages I–IV, corresponding to minimal, mild, moderate, and severe disease, respectively. The staging accounts for both the number and size of endometriotic lesions and their anatomic distribution, including genital and extragenital sites such as the bowel and appendix, providing a more comprehensive assessment of disease extent and severity [17]. When both genital and extragenital staging were documented, the more severe stage was recorded; when only one was available, that value was used. 

Statistical Analysis (R 4.3.1, 16 June 2023): Continuous variables (age, BMI) were summarized as mean ± SD and compared between high-stage (III–IV) and low-stage (I–II) groups using two-sided *t*-tests. Categorical variables (abnormal appendix, endometriotic lesions, adhesions, fibrous obliteration, inflammation, neuroendocrine tumors) were reported as n (%) within each stage group and compared using chi-square tests or Fisher’s exact tests when expected counts were small. Statistical significance was set at α = 0.05 (two-sided).

## 3. Results

Between January 2024 and April 2025, a total of 556 laparoscopic surgeries for endometriosis were performed at our center, of which 236 (42.45%) patients underwent selective appendectomy. Endometriosis was confirmed in all 236 cases. Among these, abnormal appendiceal pathology was identified in 216 patients (91.53%), while 20 patients (8.47%) had no significant pathological findings in the appendix.

Among the 236 patients, the mean age was 35.52 ± 7.04 years (range 17–57), and the mean BMI was 24.00 ± 4.17 kg/m^2^ (range 17.4–38.0). Patient demographics are summarized in Table 1.

Among the 216 patients with confirmed appendiceal abnormalities, many presented with more than one pathological finding (Figure 1, Figure 2 and Figure 3). Specifically, 34 patients (14.41%) had histologically confirmed appendiceal endometriosis lesions, 140 patients (59.32%) showed focal or serosal adhesions, 82 (34.75%) demonstrated fibrous obliteration, typically observed as obliteration of the appendiceal tip, and 20 (8.47%) exhibited signs of inflammation. Notably, three cases were diagnosed with neuroendocrine tumors of the appendix: one was confirmed to be malignant, while the other two were benign (Table 2).

The staging of endometriosis was determined intraoperatively using the classification system developed by Nezhat C. et al. [17]. Based on this system, 9 patients (3.81%) were classified as Stage I, 28 (11.86%) as Stage II, 58 (24.58%) as Stage III, and 141 (59.75%) as Stage IV (Table 1).

Table 3 compares the presence of specific appendiceal pathology between patients with high-stage (III–IV) and low-stage (I–II) endometriosis. No statistically significant differences were observed in the rates of abnormal appendix, adhesions, fibrous obliteration, or inflammation across stages. However, endometriotic lesions of the appendix showed a near-significant association with advanced-stage disease (*p* = 0.05), suggesting that appendiceal endometriosis may be more likely to occur in advanced stages. All other pathologies appeared to be independent of disease stage.

There were no intraoperative complications reported. Postoperatively, one patient required readmission two weeks after surgery for evaluation of fever. The patient was observed for 24 h and discharged in stable condition. Clinical evaluation found no direct association between the readmission and either the appendectomy or the laparoscopic procedure for endometriosis.

## 4. Discussion

Between January 2024 and April 2025, a total of 236 patients undergoing laparoscopic surgery with or without robotic assistance for endometriosis also underwent selective appendectomy. Endometriosis was confirmed in all patients Histopathologic examination revealed abnormal appendiceal pathology in 216 patients (91.53%), while 20 patients (8.47%) had no significant findings. The most common histologic abnormalities included adhesions (59.32%), fibrous obliteration (34.75%) typically affecting the appendiceal tip, and inflammation (8.47%). Endometriotic lesions within the appendix were identified in 34 cases (14.41%), and neuroendocrine tumors were diagnosed in three cases (1.27%), one of which was malignant. The majority of our patients had high-stage endometriosis (Stage III–IV). Analysis of appendiceal pathology by disease stage showed no statistically significant differences for most findings; however, appendiceal endometriosis was more frequently observed in high-stage disease. Importantly, no intraoperative complications were reported and only one patient required readmission for fever within two weeks postoperatively.

In our prior 1991 study of 100 consecutive incidental appendectomies performed during operative laparoscopy, pathologic review showed abnormal appendiceal pathology in 48% overall, comprising periappendiceal adhesions in 28%, appendiceal endometriosis in 14%, and focal chronic serosal inflammation in 4%; there were also single cases of benign mucocele (1%) and carcinoid tumor (1%) [16].

In our 2005 series of 231 women with laparoscopically confirmed endometriosis who underwent concomitant appendectomy for chronic pelvic pain, abnormal appendiceal histopathology was present in 49.8% overall, comprising appendiceal endometriosis in 22.1%, fibrous obliteration in 11.7%, serosal adhesions in 6.9%, inflammation in 3.9% (chronic appendicitis 3.0%; acute appendicitis in 0.9%), and carcinoid tumor in 1.7% [12].

Across all three series, there is consistent evidence that a substantial proportion of patients with endometriosis harbor concurrent appendiceal pathology. This consistency underscores the importance of systematic intraoperative appendiceal evaluation—and selective appendectomy—during laparoscopic surgery, even when right-lower-quadrant symptoms are absent. The observed differences in the histopathologic distribution of findings (appendiceal endometriosis, inflammatory changes, fibrous obliteration, and adhesions likely arise from differences in patient populations and case mix across the studies (e.g., disease stage, surgical indication). Importantly, all series also document a small but clinically meaningful risk of appendiceal neoplasia, reinforcing the clinical value of careful appendiceal assessment in endometriosis surgery (Table 4).

### 4.1. Physiological Purpose of the Appendix

The true function of the appendix is highly controversial. Preliminary studies suggest that the appendix serves immunological purposes attributed to the high density of lymphoid tissue and serves as the primary site for immunoglobulin production [18]. This appears redundant as other lymphoid systems such as the Peyer’s patches perform similar immunological functions [18,19]. More significant is its ability to act as a bacterial reserve to replenish the gut microbiome, particularly due to its shape, size, and evolutionary conservation. It is hypothesized that the microbiota in the appendix can be used to replenish the colon following disruption of the gut flora [18].

### 4.2. Appendiceal Cancer Rising

Recent population-based analyses show that appendiceal cancer—though rare—has risen markedly in North America, with an estimated 232% increase in malignant appendiceal tumors in the United States between 2000 and 2016 and parallel rises across histologies and age groups [20]. A U.S. birth-cohort study further demonstrates that appendiceal adenocarcinoma incidence is substantially higher among Generation X and older Millennials than among those born in earlier decades, underscoring a generational shift in risk [21]. Despite these trends, etiologies remain largely unknown, particularly for early-onset disease, and appendix-specific risk factors have not been firmly established [22]. Inferences from the broader early-onset gastrointestinal (GI) cancer literature implicate poor-quality diet, obesity, sedentary behavior, alcohol use, and microbiome perturbations as plausible contributors [23]. Histology-specific trend analyses likewise confirm rising incidence across adenocarcinoma and neuroendocrine subtypes, reinforcing the need for appendix-specific prospective studies that capture adolescent/young-adult diet, BMI trajectories, antibiotic exposures, and microbiome features with stratification by histology to clarify causal pathways [20,24].

### 4.3. Appendicitis and Endometriosis Overlap

Chronic appendicitis is a rare and often debated clinical entity characterized by prolonged or recurrent inflammation of the appendix, typically presenting with vague, intermittent right lower quadrant abdominal pain. It can be accompanied by mild gastrointestinal symptoms such as nausea, bloating, or changes in bowel habits. Unlike acute appendicitis, it lacks the classic signs of acute infection and often has normal or minimally elevated inflammatory markers, making diagnosis challenging. Imaging may reveal subtle findings such as appendiceal wall thickening or distension without overt signs of rupture. Histopathologic evaluation following appendectomy may show chronic inflammatory infiltrates, fibrosis, or lymphoid hyperplasia, supporting the diagnosis. Although controversial, increasing evidence suggests that chronic appendicitis can be a genuine source of chronic pelvic pain and should be considered in the differential diagnosis of unexplained right lower quadrant pain [10].

Chronic appendicitis can be clinically confused with endometriosis due to overlapping features such as persistent or intermittent right lower quadrant abdominal pain, nonspecific gastrointestinal symptoms, and inconclusive imaging or laboratory findings. Both conditions may present without acute signs, making diagnosis challenging. In particular, endometriosis involving the appendix or adjacent pelvic structures can mimic chronic appendicitis by causing localized inflammation, adhesions, or fibrosis. Conversely, chronic appendicitis may present with symptoms similar to cyclic pelvic pain seen in endometriosis, especially in reproductive-aged women. Intraoperative findings such as an inflamed or thickened appendix may resemble endometriotic involvement, and histopathologic examination is often required for definitive diagnosis. Notably, both conditions can coexist, further complicating clinical interpretation [8,10].

### 4.4. Appendectomy in Endometriosis

Previous literature has shown that appendiceal involvement in endometriosis is relatively uncommon but clinically significant, with reported prevalence ranging from 2.8% to 13.2% among patients undergoing surgery for pelvic endometriosis [8,25,26]. The appendix may harbor endometrial implants either superficially or involving the muscularis and serosal layers, occasionally leading to symptoms that mimic acute appendicitis or chronic right lower quadrant pain. While appendiceal endometriosis is often asymptomatic and discovered incidentally during laparoscopy, its presence has been associated with more extensive pelvic disease and gastrointestinal symptoms [26]

Several other studies have also shown that appendiceal involvement is more frequently observed in advanced stages of endometriosis. While early-stage disease (Stage I–II) rarely includes extragenital involvement, Stage III–IV endometriosis is significantly associated with appendiceal lesions, particularly in patients with deep infiltrating endometriosis (DIE) or widespread pelvic adhesions. Gustofson et al. noted that appendiceal endometriosis often coexists with extensive pelvic disease [8], and as summarized by Allahqoli et al. (2023), appendiceal involvement is more likely in advanced endometriosis—particularly when the bowel, uterosacral ligaments, or peritoneum are also affected [11].

Accordingly, our data demonstrated a total prevalence of appendiceal endometriosis involvement of 14.41%, with a higher occurrence observed in patients with advanced-stage disease. However, as shown in our patient cohort, the appendix may exhibit other pathological findings beyond endometriotic lesions. In our analysis, we also identified inflammation, adhesions, and fibrous obliteration in a significant number of patients with endometriosis. These non-endometriotic lesions may contribute to pelvic pain and could potentially explain the symptomatic improvement observed after appendectomy in select patients.

These findings may reflect the inflammatory nature of endometriosis, which can affect adjacent structures such as the appendix—even in early-stage disease—due to chronic pelvic inflammation. This highlights the importance of careful intraoperative inspection of the appendix during endometriosis surgery. When abnormalities are observed, histopathological evaluation can aid in a more comprehensive assessment of disease burden and may contribute to improved symptom management.

### 4.5. Appendiceal Pathologies in Endometriosis

In patients with endometriosis, the appendix may present with a range of pathological findings beyond the presence of endometriotic lesions. One notable feature is inflammation, which can be either chronic or mimic acute appendicitis. Histologically, this is marked by chronic inflammatory cell infiltration and fibrosis of the appendiceal wall, contributing to clinical presentations similar to acute appendicitis [8]. Another observed change is luminal fibrous obliteration, where endometrial tissue may cause partial or complete blockage of the appendiceal lumen. This fibrous obliteration is often due to fibrosis, adhesions, or submucosal implants compressing the lumen, resulting in right lower quadrant pain [27]. Additionally, adhesions are frequently reported, with the appendix tethered to nearby pelvic structures such as the ovaries, uterus, or bowel. These peri-appendiceal fibrotic bands contribute to chronic pelvic pain and can complicate laparoscopic surgical approaches [28]. Though rare, some cases exhibit neuroendocrine hyperplasia or mucinous changes as a reactive phenomenon due to chronic irritation from ectopic endometrial tissue. Collectively, these findings highlight the complex and multifaceted involvement of the appendix in endometriosis and support its consideration during surgical management [29].

In our study, no cases of mucinous changes were identified; however, three incidental findings of appendiceal neuroendocrine tumors (NETs) were observed, including one malignant case. These findings underscore the clinical value of performing selective appendectomy during laparoscopic surgery for endometriosis. Neuroendocrine tumors can present in varied morphologic forms and are frequently not detectable through intraoperative visualization alone, emphasizing the critical role of histopathological assessment. Previous studies have reported that even normal-appearing appendices may harbor intraluminal pathology, which can be easily missed without tissue analysis, potentially allowing disease progression if left in situ [30,31]. Additionally, the clinical symptoms of neuroendocrine tumors—such as vague lower abdominal pain, bloating, or discomfort—can closely mimic endometriosis, further complicating accurate preoperative diagnosis [11,12,31]. In our cohort, early detection of the malignant neuroendocrine tumors through pathology likely prevented metastasis, reinforcing the significant diagnostic and therapeutic benefit of incorporating appendectomy into the surgical management of endometriosis.

### 4.6. Limitations of Intraoperative Evaluation

Due to the diverse morphology of appendices, intraoperative evaluation alone is a weak method to assuredly evaluate and assess the state of the appendix, and visual inspection of a normal appearing appendix may miss intraluminal pathology [32]. Alternatively, an abnormal appearing appendix may result in a normal finding despite clinical suspicion, as occurred in 8.47% of our cases.

According to literature, a notable proportion of appendectomy specimens reveal a histologically normal appendix, despite clinical suspicion of appendicitis. This phenomenon, often referred to as a “negative appendectomy,” has been reported in 10% to 20% of cases, and up to 30% in specific populations such as young women and children [33,34]. Endometriosis is a significant contributor to false-positive appendectomy diagnoses, particularly in reproductive-aged women. Studies have shown that up to 13–22% of negative appendectomy cases in women are later attributed to underlying gynecologic conditions, most commonly endometriosis [35,36]. Endometriosis can mimic acute or chronic appendicitis both clinically and radiologically, as patients may present with right lower quadrant abdominal pain, rebound tenderness, nausea, and elevated inflammatory markers—symptoms indistinguishable from true appendicitis. Furthermore, chronic cyclical pelvic pain associated with endometriosis may be misinterpreted as recurrent appendicitis, especially when imaging is inconclusive. Allahqoli et al., in a systematic review, reported that appendiceal endometriosis was present in 2.67% of women admitted with acute appendicitis. In addition, appendiceal endometriosis was an incidental finding during gynecologic surgery in 7.23% of cases, further illustrating the diagnostic overlap [11]. These findings underscore the need for thorough gynecological evaluation in women with atypical or recurrent right lower quadrant pain to avoid unnecessary surgery and ensure timely diagnosis of endometriosis.

### 4.7. Complications

Postoperative complications of laparoscopic appendectomies are generally uncommon, but can include surgical site infection, wound infection, intra-abdominal abscess, ileus or bowel fibrous obliteration, or hemorrhage [31,37]. The severity and likelihood of complications tends to increase with the complexity of the appendiceal disease [38].

Laparoscopic appendectomy performed simultaneously by experienced surgeons in this cohort was proven safe and yielded no significant complications. In our series of 236, the appendectomy typically required 3–7 min; there were no intraoperative complications, and all patients were discharged within 24 h (mean hospitalization 6 h). Mean follow-up was 6 months with only minor postoperative events. One postoperative complication occurred, which was promptly identified and resolved. An especially low complication rate of 0.4% in this present study suggests a strong safety profile for the dual procedure.

### 4.8. Expert Opinion

Our findings suggest that appendiceal involvement in endometriosis is more common than previously recognized in the literature. Although histologically confirmed endometriotic lesions on the appendix were identified in only 14.41% of endometriosis cases, we frequently observed other significant pathological changes—including chronic inflammation, luminal fibrous obliteration, adhesions, and fibrosis. These alterations, even in the absence of overt endometriotic implants, may contribute substantially to the chronic pelvic pain experienced by patients with endometriosis.

Appendectomy performed during laparoscopic surgery for endometriosis can be beneficial in select patients, particularly those with right lower quadrant pain or when the appendix appears abnormal intraoperatively. Clinical overlap between appendicitis and endometriosis, including acute and chronic presentations, can further complicate diagnosis and treatment decisions. Thus, selective appendectomy may contribute to more comprehensive management, particularly in cases where the appendix is implicated in the patient’s symptomatology. However, given the risks associated with any surgical intervention, the decision should be individualized based on intraoperative findings and clinical context.

Although we do not advocate for routine prophylactic appendectomy in all patients with endometriosis, based on our findings, we strongly recommend that endometriosis surgeons perform a meticulous inspection of the appendix during laparoscopic procedures. This evaluation should be guided by our proposed Extragenital Endometriosis Staging System [17]. If any abnormality—such as induration, adhesions, congestion, or atypical morphology—is detected, appendectomy should be performed using a sensitive and skillful technique to ensure that clinically relevant pathologies are not missed.

We recommend that all endometriosis surgeons discuss the potential for appendiceal abnormalities during the preoperative consultation and obtain informed surgical consent for appendectomy if suspicious findings are encountered intraoperatively. This proactive approach facilitates timely and safe management of incidental appendiceal pathology without requiring a second procedure.

Importantly, appendectomy in this setting should be carried out by a surgeon with sufficient experience in gastrointestinal procedures or in collaboration with a gastrointestinal surgical team to optimize patient safety and outcomes.

### 4.9. Limitation and Future Directions

In this retrospective, single-center study, the high-volume enriched referral population may have inflated the observed prevalence of appendiceal pathology, limiting generalizability. Prospective, multicenter work is needed to improve the external validity of our results. Future studies should assess the long-term clinical impact of selective appendectomy performed during laparoscopic surgery for endometriosis—specifically reoperation rates, symptom recurrence, and complications. Ultimately, longitudinal investigations that incorporate pathologic and biologic correlates of appendiceal disease are required to determine whether appendectomy should be reserved for cases with intraoperative/pathologic suspicion or considered prophylactically during endometriosis surgery. Given recent increases in appendiceal cancer incidence, further research is warranted in this area as well.

## 5. Conclusions

In this study, selective appendectomy performed during laparoscopic surgery for endometriosis revealed a high prevalence of appendiceal pathology. The increase in appendiceal pathology rates compared to prior studies may reflect enhanced surgical experience in identifying appendiceal abnormalities and is interesting in the context of growing evidence of increasing appendiceal cancer rates. Surgeons must carefully weigh the potential benefits of selective appendectomy—including symptom relief, prevention of missed diagnoses, and comprehensive endometriosis management—against the inherent risks, though low, of the additional surgical procedure.

## Figures and Tables

**Table 1:** Patient Demographics and Clinical Characteristics (n = 236).

Characteristic	Value
Age (years)	35.52 ± 7.04 (17–57)
Body Mass Index (kg/m^2^)	24.0 ± 4.17 (17.4–38.0)
APP Score ^1^	
High risk	174 (73.73%)
Moderate risk	19 (8.05%)
Low risk	27 (11.44%)
Not completed	16 (6.78%)
Primary Presenting Complaint	
Pain	102 (43.22%)
Infertility	100 (42.37%)
Pain and Infertility	23 (9.75%)
Other	11 (4.66%)
Confirmed Endometriosis	236 (100%)
Stage of Endometriosis ^2^	
Stage I	9 (3.81%)
Stage II	28 (11.86%)
Stage III	58 (24.58%)
Stage IV	141 (59.75%)

^1^ APP Score: The Endometriosis Advisor APP Score is a validated symptom-based risk stratification tool that predicts the likelihood of endometriosis based on patient-reported symptoms and history. A score >90 indicates high risk, 50–90 suggests moderate risk, and <50 indicates low probability of disease. ^2^ Staging based on intraoperative assessment using the modified classification system developed by Dr. Camran Nezhat.

**Table 2:** Appendiceal Pathology Findings.

Pathologic Finding	Number of Cases	% of Total (n = 236)
Confirmed abnormal pathology ^1^	216	91.53%
Endometriosis of appendix	34	14.41%
Adhesions	140	59.32%
Fibrous obliteration	82	34.75%
Inflammation	20	8.47%
Neuroendocrine tumors (NETs)	3	1.27%

^1^ Pathologic abnormalities were identified in 216 of 236 appendectomy specimens. Percentages for individual findings are calculated based on the 236 cases with confirmed endometriosis. Some patients exhibited more than one histopathologic feature.

**Table 3:** Distribution of Appendiceal Pathologic Findings by Endometriosis Stage.

Pathologic Finding	High Stage (N = 199) n (%)	Low Stage (N = 37) n (%)	*p*-Value
Abnormal appendix	184 (92.46%)	32 (86.49%)	0.38
Endometriotic lesion	33 (16.58%)	1 (2.7%)	0.05
Adhesions	119 (59.8%)	21 (56.76%)	0.87
Fibrous obliteration	66 (33.17%)	16 (43.24%)	0.32
Inflammation	17 (8.54%)	3 (8.11%)	1.00 *

* Fisher’s Exact Test was used for small, expected cell counts in the inflammation category.

**Table 4:** Comparison of Histopathologic Findings Across Three Appendectomy Series.

Study/Period	N	Abnormal Appendix n (%)	Adhesions n (%)	Fibrous Obliteration n (%)	Inflammation n (%)	Appendiceal Endometriosis n (%)	Carcinoid/Neuroendocrine Tumor (NET) n (%)
Current series	236	216 (91.53%)	140 (59.32%)	82 (34.75%)	20 (8.47%)	34 (14.41%)	3 (1.27%)
Series (2005) [12]	231	115 (49.8%)	16 (6.9%)	27 (11.7%)	9 (3.9%)	51 (22.1%)	4 (1.7%)
Series (1991) [16]	100	48 (48%)	28 (28%)	12 (12%)	4 (4%)	14 (14%)	1 (1%)

## Data Availability

The raw data supporting the conclusions of this article will be made available by the authors on request.

## References

[1] Nezhat C.R., Stevens A., Balassiano E., Soliemannjad R. (2015). Robotic-assisted laparoscopy vs conventional laparoscopy for the treatment of advanced stage endometriosis. J. Minim. Invasive Gynecol..

[2] Nezhat C., Nezhat F., Nezhat C. (2012). Endometriosis: Ancient disease, ancient treatments. Fertil. Steril..

[3] Harada T. (2013). Dysmenorrhea and endometriosis in young women. Yonago Acta Med..

[4] Nezhat C., Khoyloo F., Tsuei A., Armani E., Page B., Rduch T., Nezhat C. (2024). The prevalence of endometriosis in patients with unexplained infertility. J. Clin. Med..

[5] Pirtea P., Cicinelli E., De Nola R., de Ziegler D., Ayoubi J.M. (2021). Endometrial causes of recurrent pregnancy losses: Endometriosis, adenomyosis, and chronic endometritis. Fertil. Steril..

[6] Nezhat C., Paka C., Gomaa M., Schipper E. (2012). Silent loss of kidney seconary to ureteral endometriosis. JSLS.

[7] Tsuei A., Nezhat F., Amirlatifi N., Najmi Z., Nezhat A., Nezhat C. (2025). Comprehensive management of bowel endometriosis: Surgical techniques, outcomes, and best practices. J. Clin. Med..

[8] Gustofson R.L., Kim N., Liu S., Stratton P. (2006). Endometriosis and the appendix: A case series and comprehensive review of the literature. Fertil. Steril..

[9] Centini G., Ginetti A., Colombi I., Cannoni A., Giorgi M., Ferreira H., Fedele F., Pacifici M., Martire F.G., Zupi E. (2024). Endometriosis of the appendix: Prevalence, associated lesions, and proposal of pathogenetic hypotheses. A retrospective cohort study with prospectively collected data. Arch Gynecol Obstet..

[10] Ljubas I., Jurca I., Grgić D. (2024). Chronic Appendicitis: Possible Differential Diagnosis in Patients with Chronic Abdominal Pain. Case Rep Surg..

[11] Allahqoli L., Mazidimoradi A., Momenimovahed Z., Günther V., Ackermann J., Salehiniya H., Alkatout I. (2023). Appendiceal endometriosis: A comprehensive review of the literature. Diagnostics.

[12] Berker B., Lashay N., Davarpanah R., Marziali M., Nezhat C.H., Nezhat C. (2005). Laparoscopic appendectomy in patients with endometriosis. J. Minim. Invasive Gynecol..

[13] Nezhat C., Armani E., Chen H.-C.C., Najmi Z., Lindheim S.R., Nezhat C. (2023). Use of the free Endometriosis Risk Advisor app as a non-invasive screening test for endometriosis in patients with chronic pelvic pain and/or unexplained infertility. J. Clin. Med..

[14] Nezhat C., Rambhatla A., Miranda-Silva C., Asiaii A., Nguyen K., Eyvazzadeh A., Tazuke S., Agarwal S., Jun S., Nezhat A. (2020). BCL-6 Overexpression as a predictor for endometriosis in patients undergoing in vitro fertilization. JSLS.

[15] Nezhat C., Nezhat F., Nezhat C. (2015). Nezhat’s Video-Assisted and Robotic-Assisted Laparoscopy and Hysteroscopy.

[16] Nezhat C., Nezhat F. (1991). Incidental appendectomy during videolaseroscopy. Am. J. Obstet. Gynecol..

[17] Nezhat C.R., Oskotsky T.T., Robinson J.F., Fisher S.J., Tsuei A., Liu B., Irwin J.C., Gaudilliere B., Sirota M., Stevenson D.K. (2025). Real world perspectives on endometriosis disease phenotyping through surgery, omics, health data, and artificial intelligence. NPJ Womens Health.

[18] Girard-Madoux M.J., de Agüero M.G., Ganal-Vonarburg S.C., Mooser C., Belz G.T., Macpherson A.J., Vivier E. (2018). The immunological functions of the Appendix: An example of redundancy?. Semin. Immunol..

[19] Kooij I.A., Sahami S., Meijer S.L., Buskens C.J. (2016). Te Velde AA. The immunology of the vermiform appendix: A review of the literature. Clin. Exp. Immunol..

[20] Singh H., Koomson A.S., Decker K.M., Park J., Demers A.A. (2020). Continued increasing incidence of malignant appendiceal tumors in Canada and the United States: A population-based study. Cancer.

[21] Holowatyj A.N., Washington M.K., Goldberg R.M., Murphy C.C. (2025). Birth cohort effects in appendiceal adenocarcinoma incidence across the United States. Ann. Intern. Med..

[22] Gibbs T., Washington M.K., Eng C., Idrees K., Davis J., Holowatyj A.N. (2021). Histologic and racial/ethnic patterns of appendiceal cancer among young patients. Cancer Epidemiol. Biomarkers Prev..

[23] Jayakrishnan T., Ng K. (2025). Early-onset gastrointestinal cancers: A review. JAMA.

[24] Wang D., Ge H., Lu Y., Gong X. (2023). Incidence trends and survival analysis of appendiceal tumors in the United States over the past 20 years. PLoS ONE.

[25] Dindo D., Demartines N., Clavien P.-A. (2004). Classification of surgical complications: A new proposal with evaluation in a cohort of 6336 patients and results of a survey. Ann. Surg..

[26] Ding D.C., Chang Y.H., Liu H.W. (2020). Appendiceal endometriosis in women with and without gastrointestinal symptoms. Taiwan. J. Obstet. Gynecol..

[27] Hislop J., Folman R., Wren B. (2005). Appendiceal endometriosis. Postgrad. Med. J..

[28] Emre A., Akbulut S., Yilmaz M., Bozdag Z. (2010). Laparoscopic appendectomy for appendiceal endometriosis presenting as acute appendicitis: A case report and review of the literature. World J. Gastroenterol..

[29] Ferrero S., Camerini G., Ragni N., Seracchioli R., Venturini P.L., Remorgida V. (2014). Deep endometriosis: The role of the appendix. Hum. Reprod. Update.

[30] Kaltsas G., Walter T., Knigge U., Toumpanakis C., Santos A.P., Begum N., Pape U.F., Volante M., Frilling A., Couvelard A. (2023). European Neuroendocrine Tumor Society (ENETS) 2023 guidance paper for appendiceal neuroendocrine tumours (aNET). J. Neu-roendocrinol..

[31] Harriott C.B., Sadava E.E. (2024). Management of complications after appendectomy: Literature review. Curr. Probl. Surg..

[32] Wie H.J., Lee J.H., Kyung M.S., Jung U.S., Choi J.S. (2008). Is incidental appendectomy necessary in women with ovarian endometrioma?. Aust. N. Z. J. Obstet. Gynaecol..

[33] Bolmers M.D.M., de Jonge J., van Rossem C.C., van Geloven A.A.W., Bemelman W.A., Snapshot Appendicitis Collaborative Study group (2020). Discrepancies between intraoperative and histological evaluation of the appendix in acute appendicitis. J. Gastrointest. Surg..

[34] Marudanayagam R., Williams G.T., Rees B.I. (2006). Review of the pathological results of 2660 appendicectomy specimens. J. Gastroenterol..

[35] Flum D.R., Morris A., Koepsell T., Dellinger E.P. (2001). Has misdiagnosis of appendicitis decreased over time? A population-based analysis. JAMA.

[36] Bickell N.A., Aufses A.H., Rojas M., Bodian C. (2006). How time affects the risk of rupture in appendicitis. J. Am. Coll. Surg..

[37] Khan M.N., Fayyad T., Cecil T.D., Moran B.J. (2007). Laparoscopic versus open appendectomy: The risk of postoperative infectious complications. JSLS.

[38] Surabhi A., Behura A., Behera C.R., Patra R.K., Panda B., Mishra A., Karnati R., Mohanty S., Behera C., Patra Sr R.K. (2023). Post-operative outcomes of laparoscopic appendectomy in acute complicated appendicitis: A single center study. Cureus.

